# Causal association between tobacco use, fibroblast growth factors, and pulmonary fibrosis: A two-sample Mendelian randomization study

**DOI:** 10.18332/tid/230999

**Published:** 2026-10-21

**Authors:** Renzhi Yu, Xiaoyue Han, Minghuan Wang, Yang Sun

**Affiliations:** 1 Department of Respiratory MedicineMudanjiang Medical University, Affiliated Hongqi HospitalMudanjiangChina; 2 Academic Affairs, Mudanjiang Medical University, Affiliated Hongqi HospitalMudanjiangChina; 3 Community Health Service Center, Mudanjiang Medical University, Affiliated Hongqi HospitalMudanjiangChina

**Keywords:** fibroblast growth factors, pulmonary fibrosis, Mendelian randomization, cigarette smoking

## Abstract

**Introduction:**

Cigarette smoking is a risk factor for pulmonary fibrosis, but its underlying mechanisms remain unclear. Fibroblast growth factors (FGFs) have been implicated in pulmonary fibrosis, yet their role in smoking-induced pulmonary fibrosis remains unclear. This study employed Mendelian randomization (MR) analysis to investigate the causal relationships among FGFs, smoking, and pulmonary fibrosis.

**Methods:**

FGFs, smoking behavior, and pulmonary fibrosis data were collected from the IEU Open GWAS Project and the GWAS Catalog. Single-nucleotide polymorphisms were used for instrumental variable selection. Inverse-variance weighted (IVW) was the primary MR method, supplemented by Weighted mode, Weighted median, and MR-Egger regression. Sensitivity analyses included MR-PRESSO, MR-Egger, Cochran’s Q, and leave-one-out.

**Results:**

IVW analysis showed that FGF10 (OR=0.74; 95% CI: 0.57–0.96, p=0.02), FGFR1 (OR=1.38; 95% CI: 1.04–1.84, p=0.03), and FGF19 (OR=0.78; 95% CI: 0.64–0.94, p=0.01) were statistically associated with pulmonary fibrosis risk. MR analysis of smoking behaviors as exposures and FGF10/FGFR1/FGF19 as outcomes showed no significant associations across all MR approaches. MR-Egger regression showed that horizontal multiplicity did not impact analysis except for FGFR3. MR-PRESSO did not identify any outliers. Sensitivity analysis showed that the association results were robust.

**Conclusions:**

FGF10, FGF19, and FGFR1 may be causally associated with pulmonary fibrosis. However, the MR analysis did not support a causal relationship between smoking and FGFs, suggesting that smoking contributes to pulmonary fibrosis independently of FGFs. This highlights the need for well-designed prospective studies to minimize bias in observational studies and for larger GWAS datasets to enhance future MR analyses.

## Introduction

Cigarette smoking remains a major global health concern, contributing to the development of respiratory diseases. Chronic exposure to cigarette smoking is a well-established risk factor for lung injury, primarily through mechanisms involving oxidative stress, inflammation, and tissue remodeling^1^. Among the smoking-related pulmonary diseases, pulmonary fibrosis, especially idiopathic pulmonary fibrosis (IPF), is of particular concern due to its progressive nature and poor prognosis^2^. However, the precise mechanisms by which smoking promotes pulmonary fibrosis remain incompletely understood.

Pulmonary fibrosis encompasses a broader group of over 200 interstitial lung diseases, characterized by inflammation and fibrotic remodeling of the alveolar interstitium^3^. Its etiology is diverse, including environmental, occupational, drug-induced, connective tissue disease-associated, and radiation-induced causes, as well as sarcoidosis^3^. The repair response following alveolar epithelial injury plays a critical role in disease progression. Various cell types, including endothelial cells, immune cells, epithelial cells, platelets, smooth muscle cells, and fibroblasts, secrete growth factors that regulate cell proliferation and tissue remodeling. Among them, fibroblasts are central to wound healing and fibrosis, mediating chemotaxis, extracellular matrix (ECM) protein synthesis, and the production of metalloproteinases and pro-fibrotic growth factors^4^.

Fibroblast growth factors (FGFs), comprising 18 ligands that signal via four receptors (FGFR1–4), are key mediators in pulmonary development, tissue repair, and fibrotic remodeling. Recent studies have shown that FGFs such as FGF2, FGF9, and FGF18 are upregulated in fibrotic lungs, but their functional roles remain incompletely defined, as FGFR inhibition has yielded inconsistent results in preclinical models^5^. Additionally, FGF23, a hormone-like FGF, has been implicated in lung fibrosis through its association with systemic inflammation, elevated levels in chronic airway diseases, and potential modulation of pulmonary remodeling via the α-Klotho–FGFR axis^6^. Despite these observations, it remains unclear whether FGFs are causally involved in pulmonary fibrosis.

Given the potential profibrotic or protective roles of FGFs, factors modulating FGF signaling, such as cigarette smoking, may influence disease risk. Smoking is a well-established environmental risk factor for pulmonary fibrosis and is known to promote oxidative stress and chronic inflammation, both of which can trigger fibroblast activation^7^. Preliminary evidence suggests that smoking may alter FGF expression^8^; however, whether smoking exerts its fibrotic effects through FGF-mediated pathways remains to be clarified.

Mendelian randomization (MR) utilizes genetic variants as instrumental variables (IVs) to infer causality while minimizing residual confounding and reverse causation inherent in observational designs^9^. In this study, we applied an MR approach leveraging large-scale GWAS summary statistics to systematically evaluate the causal associations between circulating FGFs, smoking behaviors, and pulmonary fibrosis. Our findings aim to clarify the causal involvement of FGF signaling in pulmonary fibrosis and provide mechanistic insights into smoking-related lung pathology.

## Methods

### Study design

The analysis was performed in accordance with the three assumptions of MR studies (Figure 1)^10^. To obtain valid causal inferences, all MR analyses strictly adhered to the three core instrumental variable assumptions: 1) The relevance assumption: the selected single-nucleotide polymorphisms (SNPs) as instrumental variables (IVs) are strongly associated with the exposure; 2) The independence assumption: the IVs are independent of potential confounders influencing both the exposure and outcome; and 3) The exclusion restriction assumption: the IVs affect the risk of outcome exclusively through the exposure, with no horizontal pleiotropy.

**Figure 1:**
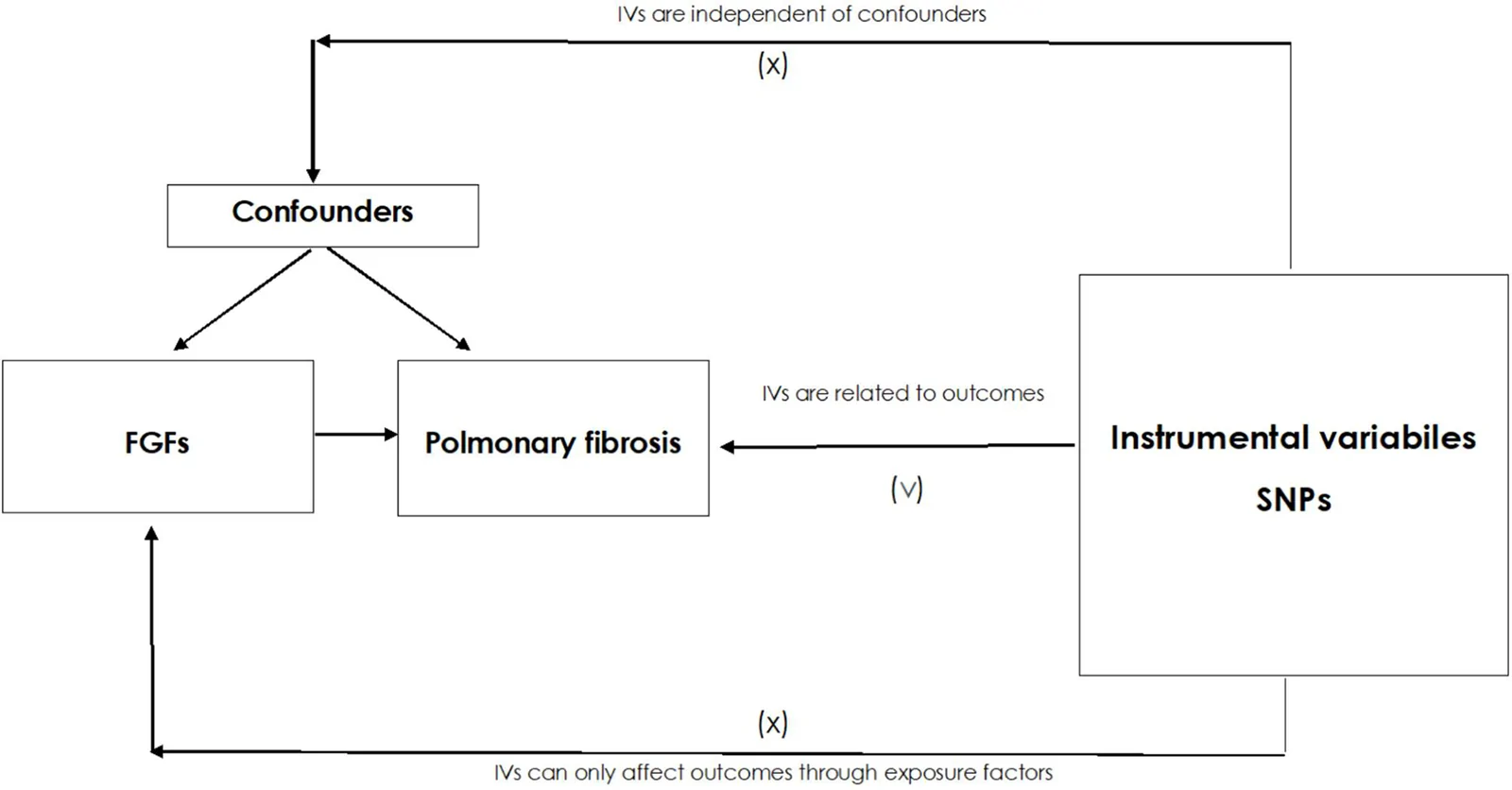
Overview of the two-sample Mendelian randomization (MR) study design

The figure provides an overview of the MR approach used to assess the causal relationship between fibroblast growth factors (FGFs) and pulmonary fibrosis. Genetic variants (single nucleotide polymorphisms, SNPs) were used as instrumental variables (IVs) to infer causality. The analysis used publicly available genome-wide association study (GWAS) summary statistics.

### Data source

Pulmonary fibrosis data (as the outcome) were extracted from the IEU Open GWAS Project based on a cross-biobank meta-analysis^10^, comprising 1566 cases and 467560 controls of European ancestry with 24195349 SNPs (GCST90018908). In that study, diagnostic definitions were harmonized across multiple cohorts (e.g. BBJ, UKB, FinnGen) using systematic mapping to ICD-10 and phecode frameworks. Pulmonary fibrosis was defined primarily by ICD-10 code J84.1 and related subcodes. FGF GWAS data were obtained from the GWAS Catalog based on the following studies: study 1 conducted a genome-wide protein quantitative trait loci analysis of 91 plasma proteins measured using the Olink Target platform in 14824 participants^11^; study 2 was a large-scale proteogenomic study involving 5368 individuals, identifying 4035 independent associations between genetic variants and 2091 serum proteins^12^.

To investigate the relationship between smoking and FGFs implicated in pulmonary fibrosis, we obtained smoking-related GWAS summary statistics from a UKB-based study^13^. Smoking status was defined using self-reported responses to UKB Field 20116, which captures current tobacco use of conventional cigarettes. Participants were categorized as: 1) never smoked (answered ‘Never’; 54520 cases and 36833 controls), 2) occasional smoking (answered ‘Only occasionally’; 1374 cases and 89979 controls); and 3) regular smoking (answered ‘Yes, on most or all days’; 2124 cases and 89229 controls). The datasets are detailed in Supplementary file Table S1.

### Ethics

This study was based exclusively on publicly available, de-identified GWAS summary statistics. No new individual-level data were collected. All original studies included in our analyses had obtained ethical approval and informed consent from participants, as documented in their respective publications. Therefore, additional ethical approval was not required for the present study.

### IV selection

FGF and smoking exposure were analyzed using single-nucleotide polymorphisms (SNPs) as IVs, which were selected based on stringent quality control criteria. SNPs associated with FGFs and never smoking were selected at genome-wide significance (p<5×10⁻^8^. Due to the limited number of strong instruments for occasional and regular smoking, SNPs associated at a relaxed threshold of p<5×10⁻^6^ were included, consistent with previous MR studies using polygenic instruments for behavioral traits^14,15^. SNPs with a minor allele frequency >0.01 were retained. Linkage disequilibrium (LD) pruning was performed using an R² threshold of <0.001 and a window size of 10000 kb^16^. If selected IVs were missing from the outcome summary data, proxy SNPs with high LD (R²>0.8) were used as replacements. For FGF–PF analyses, proxies were identified using the *TwoSampleMR* function with proxies = TRUE. For smoking–FGF analyses, proxies were manually retrieved from the 1000 Genomes EUR panel using the *LDproxy* function in the *LDlinkR* package^17^. To minimize weak instrument bias, the F-statistic was calculated for each SNP using the formula F=R²×(N-2)/(1-R²), where R² represents the proportion of exposure variance explained by the SNP. Only SNPs with an F-value >10 were retained to ensure adequate instrument strength^18^. We did not exclude SNPs based on associations with potential confounders from external databases. Instead, pleiotropic bias was assessed through MR-Egger intercepts, MR pleiotropy residual sum and outlier (MR-PRESSO), and leave-one-out sensitivity analyses.

### Data harmonization

To ensure consistency across GWAS datasets, SNP harmonization was first performed using the *harmonize_data()* function from the *TwoSampleMR* package, which aligns effect alleles and corrects strand orientation. An enhanced strategy was then applied: SNPs with extreme effect allele frequencies (EAF <0.42 or >0.58) were retained to preserve true biological signals, while those with intermediate frequencies (0.42–0.58) were excluded to minimize strand ambiguity. Complementary base pair SNPs (e.g. A/T or C/G) were manually curated and recovered when strand direction could be reliably inferred.

### MR analysis

The inverse variance weighted (IVW) method was used to assess the causal association between exposure and outcome risks, providing odds ratios (ORs) and 95% confidence intervals (CIs). To test result robustness, additional MR methods, including MR-Egger, weighted mode, simple mode, and weighted median, were applied. Analyses were performed using the ‘Two Sample MR’ package in R (version 4.3.3), and the results were visualized using scatter and sensitivity analysis plots. A p<0.05 was considered statistically significant. False discovery rate (FDR)-adjusted p-values were calculated to account for multiple comparisons. Statistical power was estimated using the mRnd web tool, incorporating sample size, variance explained by genetic instruments, and effect estimates^19^.

### Sensitivity analysis

Sensitivity analysis was performed to assess heterogeneity and pleiotropy in MR studies. Cochran’s Q test was conducted to evaluate heterogeneity among IVs, with p>0.05 indicating low heterogeneity. MR-Egger regression was used to test horizontal pleiotropy. A non-significant intercept indicates no pleiotropy. This method relies on the Instrument Strength Independent of Direct Effect (InSIDE) assumption, which posits that the pleiotropic effects of instruments are independent of their associations with the exposure. Although this assumption cannot be directly tested using summary-level data^20^, its plausibility was supported by the use of genome-wide significant SNPs, LD pruning, and the absence of significant MR-Egger intercepts. The MR-PRESSO method was used to identify outlier SNPs (p<0.05) and re-estimate causal effects after outlier removal. A leave-one-out analysis was carried out to assess the robustness of the results. All methods were applied according to current best practices in MR sensitivity analysis^21^.

## Results

### Causal association between FGFs and pulmonary fibrosis

For the analysis of FGFs as exposures and pulmonary fibrosis as the outcome, 180 FGF-related IVs were screened: 16 for FGF1, 9 for FGF10, 22 for FGF16, 15 for FGF19, 11 for FGF2, 25 for FGF21, 12 for FGF23, 11 for FGF5, 21 for FGF7, 13 for FGFR1, 8 for FGFR2, and 17 for FRFR3. F-statistics are shown in Supplementary file Table S2. Twenty-five SNPs were unavailable in the outcome summary data and lacked suitable proxy SNPs (Supplementary file Tables S2 and S3).

MR analyses suggested that FGF10 (OR=0.74; 95% CI: 0.57–0.96, p=0.02) and FGF19 (OR=0.78; 95% CI: 0.64–0.94, p=0.01) may be inversely associated with pulmonary fibrosis risk, while FGFR1 (OR=1.38 95% CI: 1.04–1.84, p=0.03) exhibited a positive association (Table 1), implying a potential contribution to disease susceptibility. The association between FGF10 and pulmonary fibrosis was consistently supported by weighted mode, weighted median, and MR-Egger regression. In contrast, associations for FGF19 and FGFR1 were supported by the weighted median analysis but showed inconsistencies in other models (Table 1), warranting cautious interpretation and further validation.

**Table 1:** Mendelian randomization (MR) analysis of the causal association between fibroblast growth factors (FGFS) and pulmonary fibrosis (outcome)

Exposure	Significance of SNP	SNPs n	Methods	OR (95% CI)	p	FDR
FGF1	5×10^-6^	16	MR Egger	1.198 (0.722–1.988)	0.496	0.8327
		16	WM	1.035 (0.742–1.442)	0.841	0.9032
		16	IVW	1.024 (0.811–1.294)	0.842	0.9258
		16	Weighted mode	1.072 (0.626–1.834)	0.804	0.9907
FGF5	5×10^-6^	11	MR Egger	1.156 (0.665–2.008)	0.619	0.8327
		11	WM	1.054 (0.787–1.413)	0.723	0.9032
		11	IVW	1.07 (0.86–1.331)	0.543	0.8539
		11	Weighted mode	1.002 (0.687–1.462)	0.991	0.9907
FGF7	5×10^-6^	20	MR Egger	1.354 (0.883–2.079)	0.182	0.8327
		20	WM	0.984 (0.752–1.287)	0.906	0.9032
		20	IVW	1.047 (0.854–1.282)	0.66	0.9071
		20	Weighted mode	0.987 (0.724–1.346)	0.936	0.9907
FGF10	5×10^-6^	9	MR Egger	0.465 (0.259–0.834)	0.037	0.4091
		9	WM	0.68 (0.507–0.913)	0.01	0.1002
		9	IVW	0.737 (0.568–0.955)	0.021	0.0976
		9	Weighted mode	0.67 (0.481–0.933)	0.045	0.443
FGF16	5×10^-6^	22	MR Egger	0.929 (0.589–1.468)	0.757	0.8327
		22	WM	1.037 (0.783–1.373)	0.801	0.9032
		22	IVW	1.026 (0.837–1.258)	0.803	0.9258
		22	Weighted mode	1.083 (0.658–1.781)	0.758	0.9907
FGF23	5×10^-6^	12	MR Egger	1.017 (0.405–2.555)	0.971	0.9713
		12	WM	0.808 (0.566–1.153)	0.24	0.5386
		12	IVW	0.892 (0.658–1.208)	0.46	0.8426
		12	Weighted mode	0.721 (0.373–1.394)	0.352	0.8318
FGFR1	5×10^-6^	13	MR Egger	1.123 (0.563–2.238)	0.749	0.8327
		13	WM	1.585 (1.076–2.335)	0.02	0.1002
		13	IVW	1.383 (1.038–1.841)	0.027	0.0976
		13	Weighted mode	1.687 (0.995–2.861)	0.076	0.443
FGF2	5×10^-6^	11	MR Egger	0.909 (0.508–1.625)	0.754	0.8327
		11	WM	1.112 (0.815–1.517)	0.505	0.788
		11	IVW	1.1 (0.875–1.383)	0.416	0.8426
		11	Weighted mode	1.061 (0.661–1.703)	0.812	0.9907
FGF19	5×10^-6^	15	MR Egger	0.903 (0.525–1.553)	0.718	0.8327
		15	WM	0.748 (0.571–0.981)	0.036	0.1212
		15	IVW	0.775 (0.641–0.936)	0.008	0.0909
		15	Weighted mode	0.734 (0.519–1.037)	0.101	0.4642
FGFR3	5×10^-6^	17	MR Egger	1.37 (0.658–2.854)	0.414	0.8327
		17	WM	0.877 (0.651–1.181)	0.387	0.6917
		17	IVW	1.008 (0.782–1.299)	0.952	0.9515
		17	Weighted mode	0.823 (0.546–1.239)	0.364	0.8318
FGFR2	5×10^-6^	7	MR Egger	1.67 (0.673–4.143)	0.319	0.8327
		7	WM	1.295 (0.816–2.056)	0.273	0.5386
		7	IVW	1.281 (0.92–1.784)	0.142	0.3906
		7	Weighted mode	1.009 (0.499–2.041)	0.981	0.9907

The analysis used genome-wide association study (GWAS) summary statistics from publicly available datasets. Pulmonary fibrosis data were extracted from the IEU Open GWAS Project, including 1566 cases and 467560 controls. Data for FGFs were obtained from two GWAS studies with sample sizes of 14824 and 5368 participants, respectively. The instrumental variables were selected based on genome-wide significance thresholds (p<5×10^-6^). Multiple MR methods were applied to assess causality, including MR-Egger, weighted median (WM), inverse variance weighted (IVW), and weighted mode. FDR: false discovery rate. Significant association at p<0.05.

MR-Egger Intercept test showed no evidence of horizontal pleiotropy, except for FGFR3, where heterogeneity was detected (Table 2). MR-PRESSO analysis identified no outliers (Table 3). Therefore, they should be interpreted cautiously and require further validation in larger samples. Given the marginal pleiotropy signal for FGFR3 (MR-PRESSO global p=0.047) and the presence of heterogeneity-driving SNPs identified by radial MR, additional outlier removal was performed to ensure robustness. After excluding four influential SNPs (rs114105810, rs12501307, rs143794056, rs145412319), the causal estimate for FGFR3 remained null, and no heterogeneity or pleiotropy was observed (Supplementary file Tables S4–S6), supporting the stability of this result. The visualization results of all MR analyses are shown in Supplementary file Figures S1–S4.

**Table 2:** Assessment of heterogeneity and pleiotropy in the Mendelian randomization (MR) analysis of pulmonary fibrosis (outcome) and fibroblast growth factors (FGFs)

Exposure	Heterogeneity		Pleiotropy	
	Q statistic (IVW)	p	MR-Egger Intercept	p
FGF1	15.521	0.415	-0.0224	0.503
FGF5	10.225	0.421	-0.0169	0.770
FGF7	26.155	0.126	-0.0497	0.199
FGF10	11.075	0.197	0.0853	0.136
FGF16	23.495	0.318	0.0195	0.639
FGF23	18.155	0.078	-0.0203	0.771
FGFR1	10.405	0.580	0.0273	0.529
FGF2	7.605	0.667	0.0273	0.501
FGF19	11.553	0.642	-0.0225	0.566
FGFR3	**26.444**	**0.048**	-0.0479	0.396
FGFR2	5.748	0.452	-0.0374	0.564

Heterogeneity was evaluated using the Q statistic from the IVW method, with the corresponding p-value indicating whether significant heterogeneity is present among instrumental variables. Pleiotropy was assessed using the MR-Egger intercept test, where the intercept and its p-value indicate the presence of directional pleiotropy (p<0.05 suggests potential pleiotropic bias). These analyses help determine the validity of the MR assumptions and the robustness of causal inferences.

**Table 3:** Mendelian randomization pleiotropy residual sum and outlier (MR-PRESSO) analysis for the causal association between fibroblast growth factors (FGFs) and pulmonary fibrosis (outcome)

Exposure	Raw		Outlier corrected		Global p	Number of outliers	Distortion p
	OR (95% CI)	p	OR (95% CI)	p			
FGFR1	1.383 (1.059–1.805)	0.035	NA	NA	0.613	NA	0.98
FGF2	1.1 (0.901–1.343)	0.373	NA	NA	0.671	NA	NA
FGF1	1.024 (0.811–1.294)	0.844	NA	NA	0.441	NA	NA
FGFR2	1.281 (0.927–1.772)	0.184	NA	NA	0.471	NA	
FGF7	1.047 (0.854–1.282)	0.665	NA	NA	0.14	NA	
FGFR3	1.008 (0.782–1.299)	0.952	NA	NA	0.047	NA	
FGF19	0.775 (0.653–0.92)	0.011	NA	NA	0.67	NA	NA
FGF16	1.026 (0.837–1.258)	0.805	NA	NA	0.295	NA	
FGF10	0.737 (0.568–0.955)	0.050	NA	NA	0.252	NA	
FGF5	1.07 (0.86–1.331)	0.557	NA	NA	0.467	NA	
FGF23	0.892 (0.658–1.208)	0.475	NA	NA	0.081	NA	

The ‘Raw’ column provides the odds ratios (OR) with 95% confidence intervals (CI) and corresponding p-values before outlier correction. The ‘Outlier corrected’ column reports results after removing detected pleiotropic outliers (if applicable). The global p-value tests for horizontal pleiotropy, while the ‘Number of outliers’ indicates the count of detected outliers. The ‘Distortion p’ value assesses whether.

### Causal association between smoking and FGF levels

Given that FGFR1, FGF10, and FGF19 demonstrated potential causal associations with pulmonary fibrosis, we investigated whether smoking behaviors influence the circulating levels of these FGFs, potentially contributing to fibrotic lung pathology. For smoking behaviors as exposures and FGFs as outcomes, 3 SNPs were used for never smoking, 14 for occasional smoking, and 7 for regular smoking. F-statistics are presented in Supplementary file Table S7. Some SNPs were unavailable in the outcome summary dataset. For never smoking, one SNP (rs113332425) was unmatched, and no proxy SNPs were available. For occasional smoking, 5 SNPs were unmatched, with proxy SNP rs10279941 replacing rs6461705. For regular smoking, 3 SNPs were unmatched, with proxy SNP rs4867413 replacing rs12658552 and rs667282 replacing rs664172 (Supplementary file Table S8).

MR analysis using IVW, MR-Egger, weighted median, and weighted mode methods found no significant associations between smoking status and circulating levels of the tested FGFs (Supplementary file Table S9), suggesting a lack of evidence for a causal influence of smoking on these FGF pathways.

Sensitivity analyses showed no evidence of heterogeneity or horizontal pleiotropy (Supplementary file Table S10). MR-PRESSO identified no significant outliers, except for the association between regular smoking and FGF19 (p=0.010), indicating potential pleiotropy (Supplementary file Table S11). Overall, the MR estimates appeared robust and unlikely to be biased by horizontal pleiotropy.

## Discussion

In this study, we employed MR to assess the potential causal relationship between FGFs and pulmonary fibrosis, as well as the role of smoking in modulating FGF-related pathways. Our findings suggested that FGF10, FGF19, and FGFR1 were significantly associated with pulmonary fibrosis, indicating their possible involvement in disease pathogenesis. The observed effect sizes (OR ≈ 0.7–1.4) were modest but biologically plausible, consistent with the polygenic architecture of pulmonary fibrosis and the expected magnitude of genetic effects in MR studies involving circulating proteins^22^. In contrast, no significant associations were observed between smoking behaviors and FGF levels, suggesting that smoking may contribute to pulmonary fibrosis through mechanisms independent of FGF signaling. Smoking has been shown to induce fibrosis through alternative pathways, including the generation of reactive oxygen species (ROS), epithelial cell apoptosis, macrophage activation, and sustained release of profibrotic cytokines such as TGF-β1 and IL-13. These processes promote ECM deposition, myofibroblast differentiation, and alveolar remodeling, all of which contribute to fibrotic progression independently of FGF signaling. Overall, our findings support a potential etiological role for FGF10, FGF19, and FGFR1 in pulmonary fibrosis, while highlighting that smoking likely acts through distinct profibrotic mechanisms.

FGFs are classified into six subfamilies based on sequence homology and functional similarities. Among them, FGF10 is a paracrine signaling molecule involved in embryonic lung development and tissue repair. Defects in FGF10 signaling are associated with neonatal lung disease, and FGF10 mutations have been linked to congenital airway abnormalities that increase susceptibility to chronic airway diseases in adulthood^23^. FGF10 prevents fibrosis by promoting the proliferation of injury-activated alveolar progenitors and enhancing alveolar repair, thereby reducing fibrotic scarring and collagen deposition in the lung^24^. Hadzic et al.^25^ have demonstrated that cigarette smoking downregulates FGF10 expression in alveolar walls, contributing to emphysema and pulmonary hypertension, whereas FGF10 overexpression reverses smoke-induced damage. However, the relationship between smoking and FGF10 in pulmonary fibrosis remains unclear. Our study identified a significant negative association between FGF10 and pulmonary fibrosis; however, smoking did not show a genetic link to FGF10. One possible explanation is that FGF10 plays a fundamental role in lung tissue regeneration, and its deficiency may contribute to fibrosis development. In contrast, smoking likely induces fibrosis through indirect mechanisms, such as oxidative stress, epithelial injury, and immune dysregulation, rather than direct modulation of FGF10.

FGF19 plays a crucial role in bile acid metabolism and lipid homeostasis. It has also been identified as an antifibrotic molecule with potential therapeutic relevance in fibrotic lung disorders. Justet et al.^26^ have found that FGF19 is often downregulated in IPF, with lower plasma concentrations observed in IPF patients compared to controls. However, previous studies have not fully addressed the issue of causality. In our MR analysis, we observed a negative association between genetically predicted FGF19 levels and pulmonary fibrosis risk, supporting a potential protective role for FGF19. Furthermore, we did not identify a significant genetic association between smoking and FGF19 levels, aligning with previous findings that smoking cessation does not alter plasma FGF19 concentrations^27^. This suggests that FGF19 regulation may be independent of smoking exposure, and smoking-related fibrosis likely develops through alternative mechanisms.

FGF/FGFR signaling is involved in the development of multiple organs, including the lung. FGFR1, a type 4 receptor tyrosine kinase, plays a crucial role in cellular migration, survival, and tissue remodeling. Altered expression of FGFR1 and FGF1 has been reported in IPF lungs, suggesting that FGFR1 signaling may contribute to fibroblast migration and fibrotic remodeling^28^. Preclinical data have also shown that pharmacological inhibition of FGFR1 (NP603) attenuates hepatic fibrosis in rats, supporting a potential profibrotic role for FGFR1^29^. In line with these findings, our MR analysis identified a positive association between genetically predicted FGFR1 levels and pulmonary fibrosis risk, supporting its possible involvement in fibrosis progression. Previous studies have reported a significant association between cigarette smoking and FGFR1 amplification in non-small cell lung cancer, and maternal smoking has been linked to FGFR1 upregulation in umbilical cord tissue, raising the possibility of smoking-induced FGFR1 dysregulation. However, our MR analysis did not detect a direct genetic association between smoking and FGFR1 in pulmonary fibrosis. This discrepancy suggests that although smoking may affect FGFR1 expression in certain pathological settings, its influence on fibrosis may be mediated through indirect or multifactorial mechanisms.

While our study prioritized FGF10, FGF19, and FGFR1 as causal contributors to pulmonary fibrosis, we did not observe a significant association for other FGFs such as FGF9, which has been reported to play a profibrotic role in animal models. For instance, FGF9 has been shown to promote fibroblast survival and migration while inhibiting epithelial repair, thereby contributing to fibrotic remodeling in IPF lungs^30^. The lack of genetic evidence for FGF9 in our MR analysis may reflect tissue-specific effects, differences in disease stage, or limited instrument strength for circulating FGF9 levels. These discrepancies highlight the complexity of FGF family signaling in fibrosis and underscore the importance of integrating genetic and experimental data when evaluating candidate targets.

Given the genetic evidence implicating FGF10 and FGF19 as protective factors and FGFR1 as a risk factor in pulmonary fibrosis, these molecules may represent potential therapeutic targets. Preclinical studies have shown that exogenous FGF10 administration can enhance alveolar repair and mitigate fibrosis^24^, while FGF19 analogs have demonstrated antifibrotic activity in other organ systems^31^. Conversely, FGFR1 inhibitors have been explored for their capacity to suppress fibroblast activation and ECM deposition^32^. Our findings support further exploration of FGF-based agonists or FGFR1-targeted antagonists as candidate therapies for pulmonary fibrosis.

Notably, for both FGF19 and FGFR1, we observed discrepancies in effect estimates across different MR models. While the IVW and weighted median methods supported a protective effect of FGF19 and a risk effect of FGFR1, these associations were not replicated in the MR-Egger and weighted mode analyses. Such inconsistencies may arise from violations of underlying assumptions specific to each method. IVW assumes all instruments are valid and unconfounded, making it sensitive to outlier SNPs or unbalanced pleiotropy. MR-Egger, though more robust to horizontal pleiotropy, requires that pleiotropic effects be independent of SNP–exposure associations (InSIDE assumption), which may not always hold. The weighted mode method is more robust to invalid instruments but assumes that the largest number of similar SNPs reflect the true causal effect, which may be violated when heterogeneity is high or instruments are weak^33^. Biologically, these inconsistencies may reflect the influence of FGF19- and FGFR1-associated SNPs on alternate pathways, such as lipid metabolism or signaling via other FGFRs, that affect pulmonary fibrosis independently of the target protein. Therefore, although the IVW results suggest a potential causal role, the lack of concordance across sensitivity analyses indicates possible residual pleiotropy or weak instruments. These findings should be interpreted cautiously and warrant further validation through functional studies or pleiotropy-robust MR frameworks.

### Limitations

Despite the strengths of MR in inferring causality and mitigating reverse causation, such as the possibility that pulmonary fibrosis may secondarily alter FGF expression, several limitations should be acknowledged. First, horizontal pleiotropy cannot be fully excluded, although MR-Egger and MR-PRESSO analyses indicated no significant pleiotropy for the main associations, except for FGFR3 and regular smoking–FGF19, which warrants cautious interpretation. Second, while we selected robust genetic instruments with F-statistics >10, residual confounding from weak instruments, particularly in smoking-related traits with limited SNPs, may influence effect estimates. Third, gene–environment interactions and sex-specific effects could not be explored, as individual-level data were unavailable. Fourth, although MR assumptions were assessed through sensitivity analyses, unobserved violations (e.g. canalization or linkage disequilibrium with unmeasured traits) may still bias results. Fifth, while proxy SNPs were selected based on high LD (R² >0.8) using *TwoSampleMR* and *LDlinkR*, functional equivalence to the original SNPs could not be validated, which may introduce exposure misclassification. Sixth, statistical power was limited for the FGF–outcome associations, warranting cautious interpretation and future validation in larger cohorts. Lastly, our analysis used predominantly European-ancestry GWAS data, which may limit generalizability. Although a recent multi-ancestry IPF meta-analysis^34^ and a Mexican cohort study of the MUC5B promoter variant^35^ illustrate progress toward broader representation, MR studies of FGF signaling in non-European populations remain scarce, highlighting the need for future validation among diverse ancestries. Genetic diversity in FGF pathways and fibrotic susceptibility across ancestries remains uncharted, emphasizing the need for larger multi-ethnic GWAS and functional validation to clarify mechanisms and strengthen causal inference.

## Conclusions

Our study provides MR evidence suggesting a negative association between FGF10 and FGF19 with pulmonary fibrosis, supporting their potential protective roles, and a positive association between FGFR1 and disease risk, indicating its possible contribution to fibrosis progression. However, despite smoking being a well-established risk factor for pulmonary fibrosis, our analysis did not identify a significant causal effect of smoking on these FGFs. This implies that smoking may promote fibrosis through alternative mechanisms, such as oxidative stress, epithelial injury, and immune dysregulation, rather than directly influencing FGF signaling. These findings emphasize the need for further research into the interplay between smoking, FGFs, and pulmonary fibrosis. Future efforts should prioritize preclinical evaluation of FGF10 and FGF19 agonists and FGFR1 inhibitors, along with the development of FGF-based biomarkers, to assess their translational potential in pulmonary fibrosis.
