# Supplementary material for: Causal association between tobacco use, fibroblast growth factors, and pulmonary fibrosis: A two-sample Mendelian randomization study
Source: Tob Induc Dis. 2026 Oct 21;24:10.18332/tid/230999. doi: 10.18332/tid/230999 (PMC13687714; doi:10.18332/tid/230999)
Supplement: Supplementary file 1 [file TID-24-190-s001.pdf]

Supplementary file

© 2026 Yu R. et al.

DOI:

10.18332/tid/230999

The content has been provided by the author(s) and has not been reviewed, verified, or endorsed by European Publishing. It may not have undergone peer review. The views, opinions, and recommendations expressed are solely those of the author(s) and do not necessarily reflect the position of European Publishing. European Publishing accepts no responsibility or liability for any consequences arising from the use of, or reliance on, this content.

Supplementary file

Supplementary Table S1. GWAS datasets for fibroblast growth factors (FGFs) and smoking exposure used in Mendelian randomization analysis. The GWAS ID corresponds to publicly available datasets. The sample sizes for FGF-related traits are provided. Smoking exposure data include case/control numbers. The number of single nucleotide polymorphisms (SNPs) used for each dataset is also indicated.

**Trait**	**Exposure factor**	**GWAS ID**	**Case/control**	**SNPs**
Fibroblast growth factor 21 levels	FGF21	GCST90274788	14,743	NA
Serum levels of protein FGF23	FGF23	GCST90088521	5,368	NA
Serum levels of protein FGF5	FGF5	GCST90088217	5356	NA
Serum levels of protein FGF1	FGF1	GCST90088414	5323	NA
Serum levels of protein FGF19	FGF19	GCST90087637	5368	NA
Serum levels of protein FGF2	FGF2	GCST90088187	5368	NA
Serum levels of protein FGF16	FGF16	GCST90088676	5357	NA
Serum levels of protein FGF7	FGF7	GCST90088712	5365	NA
Serum levels of protein FGF10	FGF10	GCST90087941	5363	NA

Serum levels of protein FGFR1	FGFR1	GCST90089070	5328	NA
Serum levels of protein FGFR3	FGFR3	GCST90087597	5361	NA
Serum levels of protein FGFR2	FGFR2	GCST90088522	5364	NA
Tobacco smoking: Never smoked	Smoking: Never smoked	ukb-d-22506_114	54,520/36,833	13,567,196
Tobacco smoking: Occasionally	Smoking: Occasional smoking	ukb-d-22506_112	1,374/89,979	9,821,991
Tobacco smoking: Smokes on most or all days	Smoking: Regular smoking	ukb-d-22506_111	2,124/89,229	10,552,527

Supplementary Table S2. F-Statistics for instrumental variables (IVs) used in Mendelian randomization analysis. This table presents the F-statistical values for instrumental variables (IVs) selected for FGFs in the MR analysis. The F-statistic assesses the strength of genetic instruments, with values above 10 indicating strong instruments that reduce the risk of weak instrument bias. The number of SNPs used for each exposure is indicated, along with the mean, maximum, and minimum F-statistics across selected IVs.

**Exposure**	**SNP**	**Mean F**	**Max F**	**Min F**
FGF1	**16**	24.09	27.18	21.35
FGF10	**9**	32.87	115.33	20.95
FGF16	**22**	22.93	29.32	20.88
FGF19	**15**	27.35	83.74	21.06
FGF2	**11**	23.36	26.59	20.88
FGF21	**25**	24.60	41.00	21.05
FGF23	**12**	23.04	28.85	20.95
FGF5	**11**	22.60	26.69	21.05
FGF7	**21**	26.57	96.28	20.93
FGFR1	**13**	23.23	28.88	20.94
FGFR2	**8**	23.30	28.91	20.93

Exposure	SNP	Mean F	Max F	Min F
FGFR3	**17**	25.78	54.84	20.91

Supplementary Table S3. Summary of instrumental variables (IVs) used in Mendelian randomization (MR) analysis of pulmonary fibrosis. This table presents the single nucleotide polymorphisms (SNPs) used as IVs in the MR analysis evaluating the causal association between fibroblast growth factors (FGFs) and pulmonary fibrosis. The table includes SNP identifiers (rsID), chromosome (chr), position (pos), effect sizes (beta.outcome), standard errors (se.outcome), outcome sample size (samplesize.outcome), P-values (pval.outcome), effect allele frequency (eaf.outcome), effect allele, other allele, and data source. SNPs were selected based on genome-wide significance thresholds ($P < 5 \times 10^{-6}$) and evaluated for their suitability as IVs based on F-statistics and pleiotropy assessments. The pulmonary fibrosis GWAS dataset for outcome data consists of 469,126 total samples (1,566 cases and 467,560 controls).

	SNP	chr	pos	beta.outcome	se.outcome	samplesize.outcome	pval	eaf.	effect_allele.outcome	other_allele.outcome	outcome
1	rs77523727	2	49591428	-0.7631	0.5255	469126	0.1465	0.003831	A	C	Pulmonary fibrosis || id:ebi-a-GCST90018908
2	rs11885376	2	53197188	0.1186	0.0517	469126	0.02165	0.107796	G	A	Pulmonary fibrosis || id:ebi-a-GCST90018908
3	rs186973231	1	28353405	0.1543	0.2117	469126	0.4661	0.0087	A	T	Pulmonary fibrosis || id:ebi-a-GCST90018908
4	rs7696681	4	137726330	-0.0217	0.036	469126	0.5457	0.376871	G	C	Pulmonary fibrosis || id:ebi-a-GCST90018908

	SNP	chr	pos	beta.outcome	se.outcome	samplesize.outcome	pval	eaf.	effect_allele.outcome	other_allele.outcome	outcome
5	rs147582986	4	47827871	0.0656	0.1393	469126	0.6376	0.01792	T	C	Pulmonary fibrosis || id:ebi-a-GCST90018908
6	rs2572329	4	48946687	0.0165	0.035	469126	0.636601	0.491361	G	T	Pulmonary fibrosis || id:ebi-a-GCST90018908
7	rs149827386	2	31531826	-0.085	0.1121	469126	0.4485	0.028767	T	G	Pulmonary fibrosis || id:ebi-a-GCST90018908
8	rs10173720	2	27855397	0.0567	0.0409	469126	0.1655	0.302102	T	A	Pulmonary fibrosis || id:ebi-a-GCST90018908
9	rs192455656	4	126869472	-0.2165	0.1666	469126	0.1936	0.013279	T	C	Pulmonary fibrosis || id:ebi-a-GCST90018908
10	rs13236741	7	77198713	0.1398	0.0749	469126	0.06185	0.062408	T	A	Pulmonary fibrosis || id:ebi-a-GCST90018908
11	rs17102508	14	65677356	-0.081	0.0526	469126	0.1239	0.104738	A	G	Pulmonary fibrosis || id:ebi-a-GCST90018908
12	rs2174753	13	69363379	0.0014	0.0353	469126	0.968	0.423865	A	T	Pulmonary fibrosis || id:ebi-a-GCST90018908

	SNP	chr	pos	beta.outcome	se.outcome	samplesize.outcome	pval	eaf.	effect_allele.outcome	other_allele.outcome	outcome
13	rs10082425	10	84459993	0.0668	0.0429	469126	0.1193	0.217252	T	C	Pulmonary fibrosis || id:ebi-a-GCST90018908
14	rs73642985	9	26727439	-0.086	0.054	469126	0.1116	0.128852	C	T	Pulmonary fibrosis || id:ebi-a-GCST90018908
15	rs150715952	9	98434575	-0.0399	0.2151	469126	0.8527	0.007682	A	C	Pulmonary fibrosis || id:ebi-a-GCST90018908
16	rs150953309	8	67073340	-0.1471	0.1288	469126	0.2533	0.020549	A	T	Pulmonary fibrosis || id:ebi-a-GCST90018908
17	rs78725927	12	83052597	-0.0567	0.0584	469126	0.3318	0.106752	C	T	Pulmonary fibrosis || id:ebi-a-GCST90018908
18	rs79676376	14	92400884	0.1214	0.0697	469126	0.081549	0.07805	A	G	Pulmonary fibrosis || id:ebi-a-GCST90018908
19	rs9305525	21	16368647	-0.0033	0.0369	469126	0.9289	0.363303	T	C	Pulmonary fibrosis || id:ebi-a-GCST90018908
20	rs118128316	16	2810624	-0.3273	0.1713	469126	0.056011	0.011694	G	T	Pulmonary fibrosis || id:ebi-a-GCST90018908

	SNP	chr	pos	beta.outcome	se.outcome	samplesize.outcome	pval	eaf.	effect_allele.outcome	other_allele.outcome	outcome
21	rs148272329	19	22113068	0.1535	0.1641	469126	0.3495	0.012898	A	C	Pulmonary fibrosis || id:ebi-a-GCST90018908
22	rs9960534	18	26966442	0.0073	0.0359	469126	0.838	0.465683	G	A	Pulmonary fibrosis || id:ebi-a-GCST90018908
23	rs78167688	18	73035631	-0.0625	0.0768	469126	0.4157	0.046403	A	T	Pulmonary fibrosis || id:ebi-a-GCST90018908
24	rs12041668	1	196663047	-0.0324	0.0412	469126	0.4317	0.260581	T	C	Pulmonary fibrosis || id:ebi-a-GCST90018908
25	rs11809413	1	114858927	-0.1717	0.1874	469126	0.3596	0.010393	G	A	Pulmonary fibrosis || id:ebi-a-GCST90018908
26	rs143566377	3	7600671	-0.2563	0.1806	469126	0.1558	0.010261	A	C	Pulmonary fibrosis || id:ebi-a-GCST90018908
27	rs115812317	3	25177902	0.3801	0.1751	469126	0.02992	0.011718	A	G	Pulmonary fibrosis || id:ebi-a-GCST90018908
28	rs79360638	3	35473861	0.0971	0.074	469126	0.1896	0.065657	C	T	Pulmonary fibrosis || id:ebi-a-GCST90018908

	SNP	chr	pos	beta.outcome	se.outcome	samplesize.outcome	pval	eaf.	effect_allele.outcome	other_allele.outcome	outcome
29	rs72656098	4	73441847	-0.4977	0.198	469126	0.01195	0.008808	G	A	Pulmonary fibrosis || id:ebi-a-GCST90018908
30	rs10034015	4	178242990	0.0523	0.0409	469126	0.2013	0.269334	A	G	Pulmonary fibrosis || id:ebi-a-GCST90018908
31	rs12239764	1	95611420	0.0183	0.0453	469126	0.6866	0.163093	G	A	Pulmonary fibrosis || id:ebi-a-GCST90018908
32	rs6555395	5	6466583	-0.1996	0.1007	469126	0.047509	0.036184	T	C	Pulmonary fibrosis || id:ebi-a-GCST90018908
33	rs114105810	5	60523920	0.3003	0.1158	469126	0.009494	0.038923	C	G	Pulmonary fibrosis || id:ebi-a-GCST90018908
34	rs11581559	1	114694322	-0.0485	0.0399	469126	0.2248	0.292245	C	T	Pulmonary fibrosis || id:ebi-a-GCST90018908
35	rs4831772	8	15714840	0.1271	0.0701	469126	0.069619	0.07866	A	G	Pulmonary fibrosis || id:ebi-a-GCST90018908
36	rs76773347	8	6720507	-0.0279	0.072	469126	0.6984	0.061436	C	T	Pulmonary fibrosis || id:ebi-a-GCST90018908

	SNP	chr	pos	beta.outcome	se.outcome	samplesize.outcome	pval	eaf.	effect_allele.outcome	other_allele.outcome	outcome
37	rs12700239	7	20865979	-0.0508	0.0442	469126	0.2502	0.17142	A	C	Pulmonary fibrosis || id:ebi-a-GCST90018908
38	rs12546999	8	70374961	-0.0097	0.0573	469126	0.8655	0.094092	A	G	Pulmonary fibrosis || id:ebi-a-GCST90018908
39	rs10992807	9	96389147	0.0104	0.0412	469126	0.8005	0.274412	G	C	Pulmonary fibrosis || id:ebi-a-GCST90018908
40	rs4427556	11	44705473	0.0254	0.0416	469126	0.5418	0.185569	T	C	Pulmonary fibrosis || id:ebi-a-GCST90018908
41	rs150619463	12	15511661	0.1067	0.2027	469126	0.5985	0.008227	T	C	Pulmonary fibrosis || id:ebi-a-GCST90018908
42	rs71481879	11	129402644	0.2973	0.2648	469126	0.2616	0.01657	A	G	Pulmonary fibrosis || id:ebi-a-GCST90018908
43	rs34378913	14	63927197	-0.0433	0.0563	469126	0.4411	0.117509	A	G	Pulmonary fibrosis || id:ebi-a-GCST90018908
44	rs12246114	10	25181900	0.1019	0.0462	469126	0.02742	0.183758	T	A	Pulmonary fibrosis || id:ebi-a-GCST90018908

	SNP	chr	pos	beta.outcome	se.outcome	samplesize.outcome	pval	eaf.	effect_allele.outcome	other_allele.outcome	outcome
45	rs2911740	8	72582203	-0.0079	0.0459	469126	0.8632	0.767687	G	A	Pulmonary fibrosis || id:ebi-a-GCST90018908
46	rs77793352	8	79607971	0.0925	0.1237	469126	0.4544	0.01757	C	T	Pulmonary fibrosis || id:ebi-a-GCST90018908
47	rs11553630	10	5959602	0.0924	0.0949	469126	0.3301	0.039001	T	C	Pulmonary fibrosis || id:ebi-a-GCST90018908
48	rs147729150	8	122941563	-1.0491	0.9727	469126	0.2808	0.001539	T	A	Pulmonary fibrosis || id:ebi-a-GCST90018908
49	rs199881215	12	52152786	-0.0999	0.3255	469126	0.759	0.005316	G	A	Pulmonary fibrosis || id:ebi-a-GCST90018908
50	rs12812339	12	4490845	0.0536	0.0389	469126	0.1679	0.232399	C	A	Pulmonary fibrosis || id:ebi-a-GCST90018908
51	rs7873138	9	110869357	0.0226	0.0392	469126	0.5644	0.224718	C	T	Pulmonary fibrosis || id:ebi-a-GCST90018908
52	rs10764758	10	129952601	0.2082	0.1605	469126	0.1946	0.985526	C	G	Pulmonary fibrosis || id:ebi-a-GCST90018908

	SNP	chr	pos	beta.outcome	se.outcome	samplesize.outcome	pval	eaf.	effect_allele.outcome	other_allele.outcome	outcome
53	rs11790037	9	130654946	0.0477	0.0447	469126	0.2857	0.175833	C	T	Pulmonary fibrosis || id:ebi-a-GCST90018908
54	rs185895488	15	38101056	-0.0029	0.2025	469126	0.9886	0.009113	T	C	Pulmonary fibrosis || id:ebi-a-GCST90018908
55	rs147092496	22	30769997	-0.0252	0.1744	469126	0.8852	0.011452	A	G	Pulmonary fibrosis || id:ebi-a-GCST90018908
56	rs5927622	X	30812106	-0.0472	0.0631	469126	0.4543	0.946156	A	G	Pulmonary fibrosis || id:ebi-a-GCST90018908
57	rs78602608	11	113851571	-0.0253	0.0708	469126	0.721099	0.072695	A	G	Pulmonary fibrosis || id:ebi-a-GCST90018908
58	rs8117150	20	42569690	0.0203	0.1554	469126	0.8961	0.014969	A	G	Pulmonary fibrosis || id:ebi-a-GCST90018908
59	rs35394067	17	65255555	-0.0544	0.0614	469126	0.3757	0.077042	T	C	Pulmonary fibrosis || id:ebi-a-GCST90018908
60	rs190116896	X	45109265	-0.0024	0.0674	469126	0.9713	0.053109	C	T	Pulmonary fibrosis || id:ebi-a-GCST90018908

	SNP	chr	pos	beta.outcome	se.outcome	samplesize.outcome	pval	eaf.	effect_allele.outcome	other_allele.outcome	outcome
61	rs138033404	X	150295554	-0.0577	0.0708	469126	0.4151	0.067768	A	G	Pulmonary fibrosis || id:ebi-a-GCST90018908
62	rs60897151	2	157869531	0.0038	0.1019	469126	0.9701	0.033609	C	T	Pulmonary fibrosis || id:ebi-a-GCST90018908
63	rs138669289	2	82959039	0.0767	0.1565	469126	0.6242	0.013431	A	T	Pulmonary fibrosis || id:ebi-a-GCST90018908
64	rs1869565	2	221731290	-0.0116	0.0468	469126	0.8048	0.201801	C	A	Pulmonary fibrosis || id:ebi-a-GCST90018908
65	rs6703668	1	242945332	-0.0742	0.0438	469126	0.09001	0.237833	A	G	Pulmonary fibrosis || id:ebi-a-GCST90018908
66	rs1568551	2	108722095	0.0472	0.0362	469126	0.1923	0.395193	T	C	Pulmonary fibrosis || id:ebi-a-GCST90018908
67	rs116203821	5	127565817	-0.0897	0.0824	469126	0.2763	0.053798	T	A	Pulmonary fibrosis || id:ebi-a-GCST90018908
68	rs12187590	5	122603613	-0.0276	0.0433	469126	0.5236	0.169791	G	A	Pulmonary fibrosis || id:ebi-a-GCST90018908

	SNP	chr	pos	beta.outcome	se.outcome	samplesize.outcome	pval	eaf.	effect_allele.outcome	other_allele.outcome	outcome
69	rs1734370	2	10978710	0.0133	0.0391	469126	0.7326	0.280888	G	A	Pulmonary fibrosis || id:ebi-a-GCST90018908
70	rs140923441	4	26349365	-0.0383	0.2058	469126	0.8522	0.008831	T	C	Pulmonary fibrosis || id:ebi-a-GCST90018908
71	rs116728888	4	65886282	0.2108	0.148	469126	0.1544	0.04956	A	G	Pulmonary fibrosis || id:ebi-a-GCST90018908
72	rs12501307	4	13540518	-0.1167	0.0575	469126	0.04227	0.827073	G	A	Pulmonary fibrosis || id:ebi-a-GCST90018908
73	rs116514483	3	131517739	0.0365	0.0768	469126	0.6346	0.059701	T	C	Pulmonary fibrosis || id:ebi-a-GCST90018908
74	rs3135835	4	1796180	0.0542	0.044	469126	0.2182	0.780228	A	G	Pulmonary fibrosis || id:ebi-a-GCST90018908
75	rs142603627	8	128833201	0.2743	0.1724	469126	0.1116	0.013095	T	C	Pulmonary fibrosis || id:ebi-a-GCST90018908
76	rs11780485	8	14242114	-0.0485	0.0987	469126	0.6233	0.036684	A	G	Pulmonary fibrosis || id:ebi-a-GCST90018908

	SNP	chr	pos	beta.outcome	se.outcome	samplesize.outcome	pval	eaf.	effect_allele.outcome	other_allele.outcome	outcome
77	rs147267295	7	97221330	-0.0175	0.1584	469126	0.9121	0.013724	A	C	Pulmonary fibrosis || id:ebi-a-GCST90018908
78	rs10225201	7	146766088	0.0293	0.0546	469126	0.5911	0.17386	C	G	Pulmonary fibrosis || id:ebi-a-GCST90018908
79	rs12681923	8	5396098	-0.053	0.0486	469126	0.2756	0.149789	A	T	Pulmonary fibrosis || id:ebi-a-GCST90018908
80	rs7307958	12	126050061	0.0569	0.0709	469126	0.4224	0.93579	C	T	Pulmonary fibrosis || id:ebi-a-GCST90018908
81	rs17298046	13	38924871	-0.1228	0.1196	469126	0.3042	0.02469	C	A	Pulmonary fibrosis || id:ebi-a-GCST90018908
82	rs76776170	14	60812419	0.0604	0.0872	469126	0.4885	0.047332	T	A	Pulmonary fibrosis || id:ebi-a-GCST90018908
83	rs139340204	14	44518296	0.0317	0.1405	469126	0.8213	0.016287	G	C	Pulmonary fibrosis || id:ebi-a-GCST90018908
84	rs2155521	11	70439153	0.0048	0.0348	469126	0.8903	0.506007	C	T	Pulmonary fibrosis || id:ebi-a-GCST90018908

	SNP	chr	pos	beta.outcome	se.outcome	samplesize.outcome	pval	eaf.	effect_allele.outcome	other_allele.outcome	outcome
85	rs1228021	11	47953142	-0.0177	0.0537	469126	0.7411	0.892325	A	G	Pulmonary fibrosis || id:ebi-a-GCST90018908
86	rs145412319	6	131940862	-0.4213	0.2011	469126	0.03618	0.008845	G	A	Pulmonary fibrosis || id:ebi-a-GCST90018908
87	rs72821213	5	165304653	0.161	0.0922	469126	0.080649	0.043882	G	A	Pulmonary fibrosis || id:ebi-a-GCST90018908
88	rs10273107	7	9381043	0.0282	0.0461	469126	0.5416	0.810498	C	A	Pulmonary fibrosis || id:ebi-a-GCST90018908
89	rs554426368	10	15500463	-0.211	0.2413	469126	0.3819	0.005899	A	G	Pulmonary fibrosis || id:ebi-a-GCST90018908
90	rs118037202	11	3980886	0.0626	0.1321	469126	0.635801	0.019867	A	G	Pulmonary fibrosis || id:ebi-a-GCST90018908
91	rs145365655	13	106805409	-0.0184	0.0998	469126	0.854	0.03422	T	G	Pulmonary fibrosis || id:ebi-a-GCST90018908
92	rs11834353	12	73160288	-0.1631	0.1853	469126	0.3786	0.007568	A	G	Pulmonary fibrosis || id:ebi-a-GCST90018908

	SNP	chr	pos	beta.outcome	se.outcome	samplesize.outcome	pval	eaf.	effect_allele.outcome	other_allele.outcome	outcome
93	rs35885684	12	94079765	0.1477	0.1226	469126	0.2286	0.021982	A	G	Pulmonary fibrosis || id:ebi-a-GCST90018908
94	rs140031694	X	37946094	-0.0696	0.0411	469126	0.090701	0.108731	T	A	Pulmonary fibrosis || id:ebi-a-GCST90018908
95	rs190856180	15	71715736	-0.0402	0.1714	469126	0.8146	0.012579	C	T	Pulmonary fibrosis || id:ebi-a-GCST90018908
96	rs10853569	18	46202233	-0.0051	0.038	469126	0.8929	0.281205	T	C	Pulmonary fibrosis || id:ebi-a-GCST90018908
97	rs17310175	20	54306047	0.0396	0.0422	469126	0.3483	0.192397	A	C	Pulmonary fibrosis || id:ebi-a-GCST90018908
98	rs148044895	20	9032914	-0.3126	0.194	469126	0.1072	0.008807	T	C	Pulmonary fibrosis || id:ebi-a-GCST90018908
99	rs3784231	14	105678906	-0.0104	0.0373	469126	0.7813	0.301826	T	A	Pulmonary fibrosis || id:ebi-a-GCST90018908
100	rs139550165	X	31548672	0.0478	0.0887	469126	0.59	0.038308	A	T	Pulmonary fibrosis || id:ebi-a-GCST90018908

	SNP	chr	pos	beta.outcome	se.outcome	samplesize.outcome	pval	eaf.	effect_allele.outcome	other_allele.outcome	outcome
101	rs601338	19	49206674	0.0555	0.0365	469126	0.1288	0.474647	A	G	Pulmonary fibrosis || id:ebi-a-GCST90018908
102	rs117523398	21	45396034	0.1914	0.1761	469126	0.2771	0.010869	T	C	Pulmonary fibrosis || id:ebi-a-GCST90018908
103	rs13090825	3	171136440	-0.0214	0.0358	469126	0.551001	0.60852	A	T	Pulmonary fibrosis || id:ebi-a-GCST90018908
104	rs2276890	4	39460490	-0.0105	0.0366	469126	0.773701	0.350075	G	C	Pulmonary fibrosis || id:ebi-a-GCST90018908
105	rs140928404	5	79384297	0.0415	0.1471	469126	0.7776	0.023917	T	G	Pulmonary fibrosis || id:ebi-a-GCST90018908
106	rs79266418	3	71939653	-0.0348	0.0756	469126	0.6453	0.093637	A	T	Pulmonary fibrosis || id:ebi-a-GCST90018908
107	rs62479839	7	97885446	0.0351	0.1022	469126	0.7311	0.032804	T	C	Pulmonary fibrosis || id:ebi-a-GCST90018908
108	rs1580180	8	59387337	-0.0462	0.0369	469126	0.2102	0.700009	C	T	Pulmonary fibrosis || id:ebi-a-GCST90018908

	SNP	chr	pos	beta.outcome	se.outcome	samplesize.outcome	pval	eaf.	effect_allele.outcome	other_allele.outcome	outcome
109	rs7850298	9	16134425	-0.0046	0.0401	469126	0.9086	0.25887	G	C	Pulmonary fibrosis || id:ebi-a-GCST90018908
110	rs113094089	13	80899665	-0.1115	0.1188	469126	0.3479	0.025894	A	T	Pulmonary fibrosis || id:ebi-a-GCST90018908
111	rs149247626	12	76107135	0.4479	0.1875	469126	0.0169	0.010423	G	A	Pulmonary fibrosis || id:ebi-a-GCST90018908
112	rs190554207	3	38938959	0.0387	0.2859	469126	0.8925	0.004452	A	G	Pulmonary fibrosis || id:ebi-a-GCST90018908
113	rs186029109	4	71281355	-0.0807	0.1444	469126	0.5761	0.017649	C	T	Pulmonary fibrosis || id:ebi-a-GCST90018908
114	rs3774862	4	5752802	0.0169	0.0354	469126	0.6338	0.485589	C	G	Pulmonary fibrosis || id:ebi-a-GCST90018908
115	rs113275209	11	75589981	0.0136	0.1106	469126	0.9024	0.04049	A	G	Pulmonary fibrosis || id:ebi-a-GCST90018908
116	rs58645950	11	1718492	0.0132	0.1076	469126	0.9027	0.029338	T	G	Pulmonary fibrosis || id:ebi-a-GCST90018908

	SNP	chr	pos	beta.outcome	se.outcome	samplesize.outcome	pval	eaf.	effect_allele.outcome	other_allele.outcome	outcome
117	rs140331656	10	126898610	0.1951	0.2281	469126	0.3924	0.006342	T	C	Pulmonary fibrosis || id:ebi-a-GCST90018908
118	rs117579134	14	48254555	-0.1775	0.1135	469126	0.1178	0.027629	A	G	Pulmonary fibrosis || id:ebi-a-GCST90018908
119	rs12050709	15	50359224	0.0057	0.0403	469126	0.8885	0.263063	C	T	Pulmonary fibrosis || id:ebi-a-GCST90018908
120	rs704	17	26694861	0.0763	0.0353	469126	0.03059	0.543684	A	G	Pulmonary fibrosis || id:ebi-a-GCST90018908
121	rs118146756	18	40487807	-0.2538	0.1385	469126	0.066881	0.020928	A	G	Pulmonary fibrosis || id:ebi-a-GCST90018908
122	rs572736373	17	16384398	0.1074	0.1571	469126	0.4942	0.010298	T	C	Pulmonary fibrosis || id:ebi-a-GCST90018908
123	rs145634723	X	152662024	0.036	0.0625	469126	0.5646	0.062422	T	C	Pulmonary fibrosis || id:ebi-a-GCST90018908
124	rs117619130	18	56483675	-0.0863	0.0992	469126	0.3842	0.03694	C	T	Pulmonary fibrosis || id:ebi-a-GCST90018908

	SNP	chr	pos	beta.outcome	se.outcome	samplesize.outcome	pval	eaf.	effect_allele.outcome	other_allele.outcome	outcome
125	rs143794056	12	62852386	-0.2789	0.1568	469126	0.075291	0.017633	A	G	Pulmonary fibrosis || id:ebi-a-GCST90018908
126	rs17670091	17	33977916	0.207	0.1692	469126	0.2211	0.0119	C	T	Pulmonary fibrosis || id:ebi-a-GCST90018908
127	rs11871079	17	37345020	-0.0179	0.1094	469126	0.8704	0.032765	A	G	Pulmonary fibrosis || id:ebi-a-GCST90018908
128	rs71350477	18	11943598	0.1805	0.097	469126	0.06279	0.036539	A	G	Pulmonary fibrosis || id:ebi-a-GCST90018908
129	rs28643543	4	1747523	0.0525	0.0395	469126	0.184	0.237116	A	G	Pulmonary fibrosis || id:ebi-a-GCST90018908
130	rs150816167	1	179571862	0.0338	0.0849	469126	0.690501	0.048011	C	T	Pulmonary fibrosis || id:ebi-a-GCST90018908
131	rs139006129	1	157994143	0.0682	0.1296	469126	0.5989	0.028894	G	A	Pulmonary fibrosis || id:ebi-a-GCST90018908
132	rs13017945	2	119344750	0.024	0.0442	469126	0.5873	0.205987	T	C	Pulmonary fibrosis || id:ebi-a-GCST90018908

	SNP	chr	pos	beta.outcome	se.outcome	samplesize.outcome	pval	eaf.	effect_allele.outcome	other_allele.outcome	outcome
133	rs7521493	1	15046245	0.0093	0.035	469126	0.7893	0.524811	A	G	Pulmonary fibrosis || id:ebi-a-GCST90018908
134	rs1121628	5	125005381	0.0506	0.0438	469126	0.248	0.827279	C	G	Pulmonary fibrosis || id:ebi-a-GCST90018908
135	rs55676299	4	95026205	0.1011	0.0859	469126	0.2391	0.038379	A	T	Pulmonary fibrosis || id:ebi-a-GCST90018908
136	rs1711396	4	179013250	0.1468	0.0548	469126	0.007428	0.135906	C	T	Pulmonary fibrosis || id:ebi-a-GCST90018908
137	rs10868292	9	88044756	0.0042	0.0406	469126	0.9166	0.239906	T	C	Pulmonary fibrosis || id:ebi-a-GCST90018908
138	rs80264659	9	93438589	0.1391	0.1865	469126	0.456	0.009621	A	T	Pulmonary fibrosis || id:ebi-a-GCST90018908
139	rs10973688	9	38174423	0.0961	0.0665	469126	0.1485	0.121298	T	C	Pulmonary fibrosis || id:ebi-a-GCST90018908
140	rs73105859	12	48142026	-0.0458	0.0561	469126	0.4144	0.107109	A	C	Pulmonary fibrosis || id:ebi-a-GCST90018908

	SNP	chr	pos	beta.outcome	se.outcome	samplesize.outcome	pval	eaf.	effect_allele.outcome	other_allele.outcome	outcome
141	rs79213950	12	23626710	-0.0471	0.096	469126	0.624001	0.046439	G	A	Pulmonary fibrosis || id:ebi-a-GCST90018908
142	rs143646229	4	176960511	1.00E-04	0.1436	469126	0.9993	0.017726	G	A	Pulmonary fibrosis || id:ebi-a-GCST90018908
143	rs7011589	8	138421884	-0.0408	0.0563	469126	0.4686	0.134058	G	A	Pulmonary fibrosis || id:ebi-a-GCST90018908
144	rs4634647	8	139642798	-0.0056	0.0895	469126	0.9504	0.073223	G	C	Pulmonary fibrosis || id:ebi-a-GCST90018908
145	rs116488543	7	132804896	0.0631	0.083	469126	0.4476	0.051395	C	T	Pulmonary fibrosis || id:ebi-a-GCST90018908
146	rs75946261	7	8276149	0.1416	0.1415	469126	0.317	0.016847	C	T	Pulmonary fibrosis || id:ebi-a-GCST90018908
147	rs6905269	6	52555293	-0.203	0.2428	469126	0.4031	0.006131	A	G	Pulmonary fibrosis || id:ebi-a-GCST90018908
148	rs75049008	7	5146375	0.0147	0.081	469126	0.8561	0.053797	T	C	Pulmonary fibrosis || id:ebi-a-GCST90018908

	SNP	chr	pos	beta.outcome	se.outcome	samplesize.outcome	pval	eaf.	effect_allele.outcome	other_allele.outcome	outcome
149	rs143016300	10	23029300	0.1248	0.2562	469126	0.6261	0.005214	T	G	Pulmonary fibrosis || id:ebi-a-GCST90018908
150	rs11160684	14	28365943	-0.107	0.0655	469126	0.1022	0.169961	C	T	Pulmonary fibrosis || id:ebi-a-GCST90018908
151	rs6638337	X	134390856	0.0154	0.0321	469126	0.632	0.288566	T	C	Pulmonary fibrosis || id:ebi-a-GCST90018908
152	rs995875	11	88512713	-0.1943	0.0827	469126	0.01878	0.048007	C	G	Pulmonary fibrosis || id:ebi-a-GCST90018908
153	rs143036127	16	63701393	0.0585	0.0988	469126	0.5538	0.035767	C	T	Pulmonary fibrosis || id:ebi-a-GCST90018908
154	rs112248293	15	61500352	-0.1806	0.1095	469126	0.099161	0.028334	A	G	Pulmonary fibrosis || id:ebi-a-GCST90018908
155	rs2375859	20	61379066	0.0234	0.0755	469126	0.7563	0.049633	G	A	Pulmonary fibrosis || id:ebi-a-GCST90018908

Supplementary Table S4. Mendelian randomization results for associations between fibroblast growth factors (FGFs) and pulmonary fibrosis after outlier removal. This table reports the odds ratios (ORs) and 95% confidence intervals (CIs) for the associations between genetically predicted levels of FGFs and pulmonary fibrosis using four MR methods: inverse variance weighted (IVW), MR-Egger, weighted median, and weighted mode. The number of instrumental SNPs (nSNP), P-values, and false discovery rate (FDR)–adjusted P-values are provided. Results were derived after removal of outliers identified by radial MR. Four heterogeneity-driving SNPs (rs114105810, rs12501307, rs143794056, rs145412319) were excluded for FGFR3.

Exposure	Outcome	nSNP	Method	OR (95% CI)	P-value	FDR
FGFR3	Pulmonary fibrosis	13	Inverse variance weighted	0.8881 (0.7157 - 1.1021)	0.2813	0.6188
			MR Egger	1.0845 (0.5544 - 2.1214)	0.817	0.8987
			Weighted median	0.849 (0.6328 - 1.139)	0.2748	0.5039
			Weighted mode	0.7054 (0.4522 - 1.1004)	0.1499	0.4122

Supplementary Table S5. Sensitivity analyses for the association between FGFR3 and pulmonary fibrosis after outlier removal. This table presents the results of heterogeneity and horizontal pleiotropy assessments for the Mendelian randomization analysis of FGFR3 and pulmonary fibrosis following outlier removal. Cochran's Q statistic (IVW) and its corresponding P value are shown to evaluate heterogeneity among the instrumental variables. Horizontal pleiotropy was assessed using the MR-Egger intercept and its P value.

Exposure	Outcome	Heterogeneity		Pleotropy	
		Q statistic (IVW)	P value	MR-Egger Intercept	P value
FGFR3	Pulmonary fibrosis	6.8924	0.8646	-2.97E-02	0.5502

Supplementary Table S6. MR-PRESSO global test and outlier-corrected estimates for the FGFR3–pulmonary fibrosis association. This table summarizes the MR-PRESSO results for detecting horizontal pleiotropy and correcting outlier effects in the Mendelian randomization analysis of FGFR3 and pulmonary fibrosis. The raw (uncorrected) causal estimates include the odds ratio (OR) with 95% confidence interval (CI) and P value. The global test P value assesses the presence of significant horizontal pleiotropy. Outlier-corrected estimates are shown when applicable.

Exposure	Outcome	Raw		Outlier-corrected		Global Test Pvalue	Outlier	Distortion_P
		OR (95% CI)	P	OR (95% CI)	P			
FGFR3	Pulmonary fibrosis	0.8881 (0.7541 - 1.0460)	0.1806	NA (NA - NA)	NA	0.869	NA	NA

Supplementary Table S7. F-Statistics for instrumental variables (IVs) used in Mendelian randomization (MR) analysis of smoking exposure. This table presents the F-statistical values for IVs related to smoking behaviors in the MR analysis. The F-statistic assesses the strength of genetic instruments, with values above 10 indicating strong instruments that minimize weak instrument bias. The number of SNPs used for each smoking exposure category and the mean, maximum, and minimum F-statistics are indicated across selected IVs. Smoking behaviors include "Never Smoking", "Occasional Smoking", and "Regular Smoking".

Exposure	SNP	Mean F	Max F	Min F
Never smoking	**3**	44.15826	82.89377	30.86675
Occasional smoking	**14**	23.07046	29.92527	21.19855
Regular smoking	**7**	23.38536	28.22575	20.88954

Supplementary Table S8. Instrumental Variable (IV) strength and matching for smoking exposure and fibroblast growth factors (FGFs) in Mendelian randomization (MR) analysis. This table presents the characteristics of IVs used in the MR analysis assessing the causal relationship between smoking behaviors and FGFs. The number of IVs (N.IVs), mean, minimum, and maximum F-statistics are reported to determine instrument strength, with $F > 10$ indicating strong instruments. The R^2 value represents the proportion of variance explained by the IVs. The "Unmatched" column lists IVs without a match in the outcome dataset, while "Proxy SNP" indicates replacements used for unmatched SNPs. The analysis includes smoking behaviors categorized as "Never Smoked", "Occasionally Smokes", and "Smokes on Most or All Days".

Exposure	Outcome	N.IVs	mean.IV.F	min.IV.F	max.IV.F	R2	Unmatched
Tobacco smoking: Smokes on most or all days	FGFR1	10	23.385359	20.889541	28.225754	0.0025593	rs76446693,rs544971983,rs4999739
Tobacco smoking: Smokes on most or all days	FGF19	10	23.385359	20.889541	28.225754	0.0025593	rs76446693,rs544971983,rs4999739
Tobacco smoking: Smokes on most or all days	FGF10	10	23.385359	20.889541	28.225754	0.0025593	rs76446693,rs544971983,rs4999739
Tobacco smoking: Occasionally	FGFR1	19	23.070457	21.198553	29.925271	0.0047972	rs186167918,rs58695394,rs184425623,rs77349979,rs118080387
Tobacco smoking: Occasionally	FGF19	19	23.070457	21.198553	29.925271	0.0047972	rs186167918,rs58695394,rs184425623,rs77349979,rs118080387
Tobacco smoking: Occasionally	FGF10	19	23.070457	21.198553	29.925271	0.0047972	rs186167918,rs58695394,rs184425623,rs77349979,rs118080387
Tobacco smoking: Never smoked	FGFR1	5	44.158264	30.866749	82.893768	0.0024156	rs113332425
Tobacco smoking: Never smoked	FGF19	5	44.158264	30.866749	82.893768	0.0024156	rs113332425

Exposure	Outcome	N.IVs	mean.IV.F	min.IV.F	max.IV.F	R2	Unmatched
Tobacco smoking: Never smoked	FGF10	5	44.158264	30.866749	82.893768	0.0024156	rs113332425

Supplementary Table S9. Mendelian randomization (MR) analysis of the causal association between smoking behaviors and fibroblast growth factors (FGFs). This table presents the results of the two-sample MR analysis assessing the causal relationship between smoking behaviors and FGFs (FGF10, FGF19, and FGFR1). The analysis used genetic instruments selected from genome-wide association studies (GWAS). Smoking behaviors are categorized as "Never Smoked", "Occasionally Smokes", and "Smokes on Most or All Days". Multiple MR methods were applied to assess causality, including inverse variance weighted (IVW), MR-Egger, weighted median, and weighted mode. The number of single nucleotide polymorphisms (N.SNPs) used for each analysis is indicated. Results are reported as odds ratios (OR) with 95% confidence intervals (CI) and corresponding P-values. A significant association ($P < 0.05$) suggests a potential causal link between smoking exposure and FGF-related traits.\

Exposure	Outcome	N.SNPs	Methods	OR (95% CI)	P	FDR
Tobacco smoking: Never smoked	FGF10	3	Inverse variance weighted	1.4718 (0.3192 - 6.7866)	0.6202	0.9603
Tobacco smoking: Never smoked	FGF10	3	MR Egger	41984.5902 (0 - 27604198319990128)	0.5836	0.9591
Tobacco smoking: Never smoked	FGF10	3	Weighted median	1.6012 (0.257 - 9.9765)	0.614	0.9136
Tobacco smoking: Never smoked	FGF10	3	Weighted mode	1.7805 (0.2162 - 14.6614)	0.6454	0.9171
Tobacco smoking: Never smoked	FGF19	3	Inverse variance weighted	2.4205 (0.4823 - 12.149)	0.2828	0.8781
Tobacco smoking: Never smoked	FGF19	3	MR Egger	14123.5505 (0 - 260319660291421312)	0.6498	0.9591
Tobacco smoking: Never smoked	FGF19	3	Weighted median	2.9961 (0.4133 - 21.717)	0.2776	0.9136

Tobacco smoking: Never smoked	FGF19	3	Weighted mode	3.9687 (0.38 - 41.4496)	0.3686	0.9171
Tobacco smoking: Never smoked	FGFR1	3	Inverse variance weighted	0.8172 (0.2326 - 2.871)	0.7528	0.9603
Tobacco smoking: Never smoked	FGFR1	3	MR Egger	0.48 (0 - 2483440882.3541)	0.9591	0.9591
Tobacco smoking: Never smoked	FGFR1	3	Weighted median	0.7157 (0.1723 - 2.9727)	0.6452	0.9136
Tobacco smoking: Never smoked	FGFR1	3	Weighted mode	0.6533 (0.1193 - 3.5764)	0.6721	0.9171
Tobacco smoking: Occasionally	FGF10	14	Inverse variance weighted	0.5488 (0.0064 - 46.7222)	0.7913	0.9603
Tobacco smoking: Occasionally	FGF10	14	MR Egger	6e-04 (0 - 14.1869)	0.1754	0.7895
Tobacco smoking: Occasionally	FGF10	14	Weighted median	0.6685 (0.0037 - 122.4223)	0.8796	0.9136
Tobacco smoking: Occasionally	FGF10	14	Weighted mode	0.0861 (0 - 3999.194)	0.6621	0.9171
Tobacco smoking: Occasionally	FGF19	14	Inverse variance weighted	0.9991 (0.0172 - 57.9814)	0.9996	0.9996
Tobacco smoking: Occasionally	FGF19	14	MR Egger	0.1191 (0 - 2201.955)	0.6788	0.9591
Tobacco smoking: Occasionally	FGF19	14	Weighted median	1.3214 (0.0088 - 197.9163)	0.9132	0.9136
Tobacco smoking: Occasionally	FGF19	14	Weighted mode	0.0022 (0 - 20.6461)	0.2129	0.9171
Tobacco smoking: Occasionally	FGFR1	14	Inverse variance weighted	1.303 (0.0783 - 21.6893)	0.8536	0.9603

Tobacco smoking: Occasionally	FGFR1	14	MR Egger	215.621 (0.2937 - 158319.9672)	0.1365	0.7895
Tobacco smoking: Occasionally	FGFR1	14	Weighted median	2.1354 (0.047 - 97.0637)	0.6968	0.9136
Tobacco smoking: Occasionally	FGFR1	14	Weighted mode	1.5202 (0.0014 - 1661.3055)	0.9084	0.9171
Tobacco smoking: Smokes on most or all days	FGF10	7	Inverse variance weighted	8.4344 (0.1589 - 447.7854)	0.2927	0.8781
Tobacco smoking: Smokes on most or all days	FGF10	7	MR Egger	5.09 (2e-04 - 112410.7488)	0.7627	0.9591
Tobacco smoking: Smokes on most or all days	FGF10	7	Weighted median	6.2236 (0.0385 - 1007.1913)	0.4811	0.9136
Tobacco smoking: Smokes on most or all days	FGF10	7	Weighted mode	3.3874 (0.0031 - 3730.1063)	0.7444	0.9171
Tobacco smoking: Smokes on most or all days	FGF19	7	Inverse variance weighted	0.1046 (0.0016 - 6.9178)	0.2911	0.8781
Tobacco smoking: Smokes on most or all days	FGF19	7	MR Egger	0.3723 (0 - 14314.7944)	0.8617	0.9591
Tobacco smoking: Smokes on most or all days	FGF19	7	Weighted median	0.0999 (7e-04 - 13.9342)	0.3605	0.9136
Tobacco smoking: Smokes on most or all days	FGF19	7	Weighted mode	0.1456 (2e-04 - 123.5614)	0.5957	0.9171
Tobacco smoking: Smokes on most or all days	FGFR1	7	Inverse variance weighted	2.1316 (0.0814 - 55.8366)	0.6496	0.9603
Tobacco smoking: Smokes on most or all days	FGFR1	7	MR Egger	0.7771 (2e-04 - 2891.2496)	0.9544	0.9591
Tobacco smoking: Smokes on most or all days	FGFR1	7	Weighted median	1.8181 (0.031 - 106.5459)	0.7735	0.9136

Tobacco smoking: Smokes on most or all days	FGFR1	7	Weighted mode	1.3589 (0.0058 - 319.1741)	0.9159	0.9171

Supplementary Table S10. Heterogeneity and pleiotropy analysis in Mendelian randomization (MR) of smoking behaviors and fibroblast growth factors (FGFs). This table presents the heterogeneity and pleiotropy analyses conducted as part of the two-sample MR study assessing the causal relationship between smoking behaviors and FGFs (FGF10, FGF19, and FGFR1). Heterogeneity was evaluated using the Q statistic from the inverse variance weighted (IVW) method. The corresponding H.P value indicates whether significant heterogeneity is present among instrumental variables. Pleiotropy was assessed using the MR-Egger intercept. The intercept and its P.P. value indicate the presence of directional pleiotropy (P < 0.05 suggests potential pleiotropic bias). These analyses help determine the validity of the MR assumptions and the robustness of causal inferences.

**Exposure**	**Outcome**	**Heterogeneity Test**		**Heterogeneity Test**	
		**Q statistic (IVW)**	**P value**	**MR-Egger Intercept**	**P value**
Tobacco smoking: Never smoked	FGF10	0.5790874	0.748605071	-0.153627	0.5943991
Tobacco smoking: Never smoked	FGF19	1.4818405	0.476675063	-0.129861	0.6763157
Tobacco smoking: Never smoked	FGFR1	0.1853681	0.911481455	0.0079681	0.9702919
Tobacco smoking: Occasionally	FGF10	21.95727	0.056027394	0.035954	0.1694704
Tobacco smoking: Occasionally	FGF19	16.450457	0.225658205	0.0113363	0.6474832
Tobacco smoking: Occasionally	FGFR1	11.165593	0.596947562	-0.02723	0.1193035
Tobacco smoking: Smokes on most or all days	FGF10	2.7113436	0.844105614	0.0030244	0.9183249
Tobacco smoking: Smokes on most or all days	FGF19	0.4944192	0.997905753	-0.007604	0.8075082
Tobacco smoking: Smokes on most or all days	FGFR1	1.3990041	0.965918972	0.006043	0.8036943

Supplementary Table S11. Mendelian randomization pleiotropy residual sum and outlier (MR-PRESSO) analysis for the causal association between smoking behaviors and fibroblast growth factors (FGFs). This table presents the results of the MR-PRESSO analysis, which was conducted to detect and correct for horizontal pleiotropy in the MR study assessing the causal relationship between smoking behaviors and FGFs (FGF10, FGF19, and FGFR1). The "R.OR (CI%)" and "R.P" columns report the odds ratios (OR) and P-values before outlier correction. The "O.OR (CI%)" and "O.P" columns indicate results after the removal of detected pleiotropic outliers (if applicable). The Global P-value tests for horizontal pleiotropy, while the Number of Outliers indicates the count of detected outliers. The Distortion P-value assesses whether the causal estimates significantly change after outlier correction. A P < 0.05 in the global test suggests potential pleiotropy

Exposure	Outcome	R.OR (CI%)	R.P	O.OR (CI%)	O.P	Global P	Number of outliers	Distortion P
Tobacco smoking: Never smoked	FGF10	0.7566 (0.4583 - 1.2489)	0.282012101	NA (NA - NA)	NA	0.287	NA	NA
Tobacco smoking: Never smoked	FGF19	1.2814 (0.8273 - 1.9849)	0.273525338	NA (NA - NA)	NA	0.840333333	NA	NA
Tobacco smoking: Never smoked	FGFR1	1.1832 (0.7978 - 1.7547)	0.407917291	NA (NA - NA)	NA	0.422666667	NA	NA
Tobacco smoking: Occasionally	FGF10	0.5488 (0.0064 - 46.7222)	0.795459219	NA (NA - NA)	NA	0.050333333	NA	NA
Tobacco smoking: Occasionally	FGF19	0.9991 (0.0172 - 57.9814)	0.99964303	NA (NA - NA)	NA	0.231	NA	NA
Tobacco smoking: Occasionally	FGFR1	1.303 (0.0962 - 17.6524)	0.845292217	NA (NA - NA)	NA	0.597	NA	NA

Tobacco smoking: Smokes on most or all days	FGF10	8.4344 (0.5841 - 121.8032)	0.168561362	NA (NA - NA)	NA	0.865333333	NA	NA
Tobacco smoking: Smokes on most or all days	FGF19	0.1046 (0.0314 - 0.3483)	0.010359317	NA (NA - NA)	NA	0.999333333	NA	NA
Tobacco smoking: Smokes on most or all days	FGFR1	2.1316 (0.4404 - 10.3163)	0.383131748	NA (NA - NA)	NA	0.965	NA	NA

.

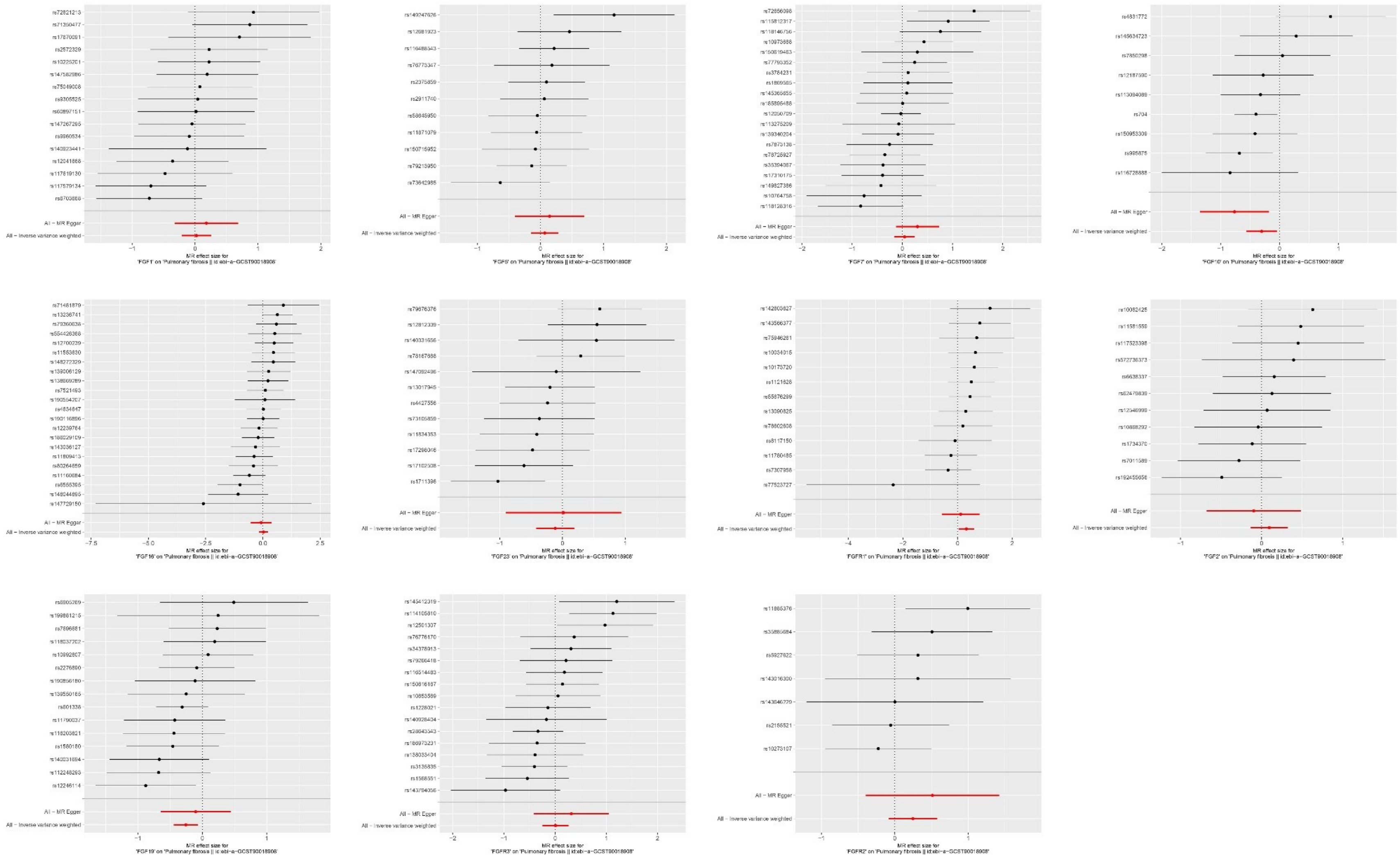

Supplementary Figure S1. Forest plot of the causal association between fibroblast growth factors (FGFs) and pulmonary fibrosis. This

figure presents the forest plots from the Mendelian randomization (MR) analysis evaluating the causal relationship between various FGFs and pulmonary fibrosis. The analysis was conducted using genome-wide association study (GWAS) summary statistics. Individual single nucleotide polymorphism (SNP) estimates and overall MR effect sizes are displayed and calculated using inverse variance-weighted (IVW) and MR-Egger methods. The forest plots illustrate the causal estimates for FGF1, FGF5, FGF7, FGF16, FGF23, FGF2, FGFR3, and FGFR2.

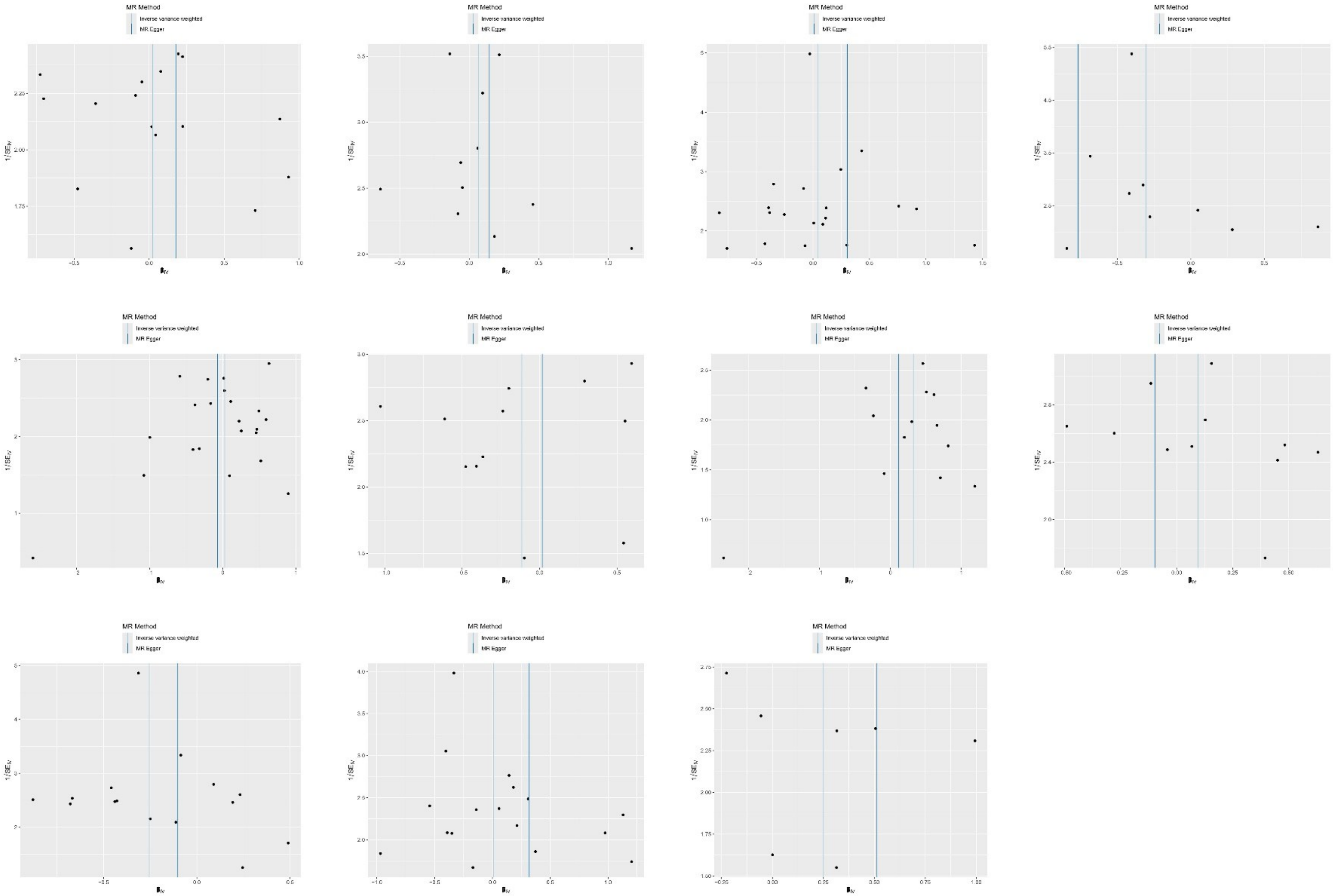

Supplementary Figure S2. Funnel plot of the causal association between fibroblast growth factors (FGFs) and pulmonary fibrosis. This figure presents the funnel plots from the Mendelian randomization (MR) analysis evaluating the causal relationship between various FGFs and

pulmonary fibrosis. The funnel plot assesses heterogeneity and potential directional pleiotropy among instrumental variables (IVs). Each dot represents a single nucleotide polymorphism (SNP) used as an instrument, with the x-axis showing the SNP effect estimates and the y-axis representing the inverse standard error (precision). Vertical lines indicate estimates from inverse variance weighted (IVW) and MR-Egger methods. Funnel plots are shown for FGF1, FGF5, FGF7, FGF16, FGF23, FGF2, FGFR3, and FGFR2.

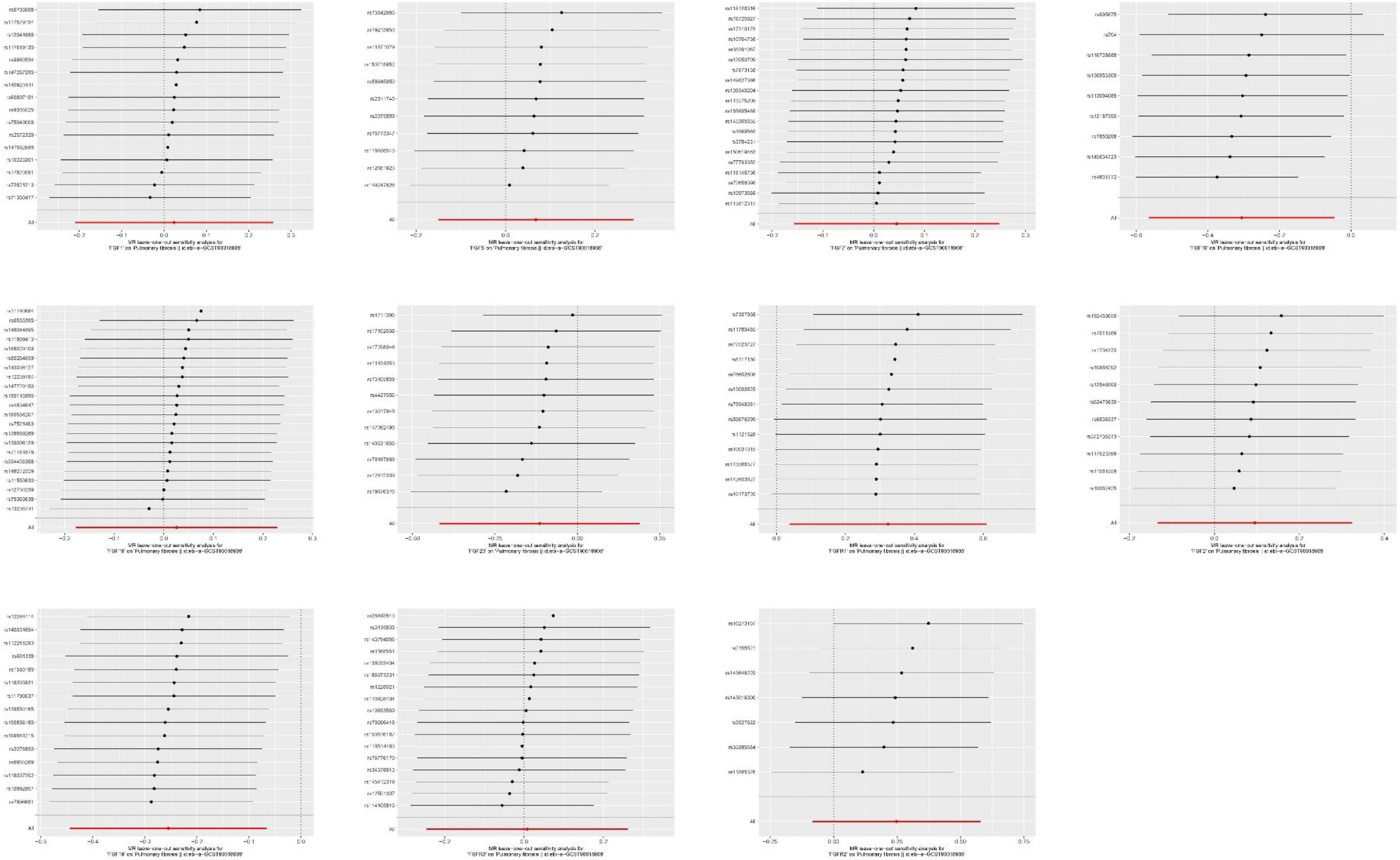

Supplementary Figure S3. Leave-one-out (LOO) sensitivity analysis for the causal association between fibroblast growth factors (FGFs) and pulmonary fibrosis. This figure presents the leave-one-out (LOO) sensitivity analysis from the Mendelian randomization (MR) study

evaluating the causal relationship between FGFs and pulmonary fibrosis. The LOO analysis assesses the robustness of the MR estimates by systematically removing one single nucleotide polymorphism (SNP) at a time and re-estimating the causal effect. The x-axis represents the effect size for each SNP. The y-axis lists the SNPs used as instrumental variables. LOO plots are shown for FGF1, FGF5, FGF7, FGF16, FGF23, FGF2, FGFR3, and FGFR2. A consistent causal estimate across SNP exclusions suggests that no single SNP disproportionately influences the results, indicating robustness in the MR analysis.

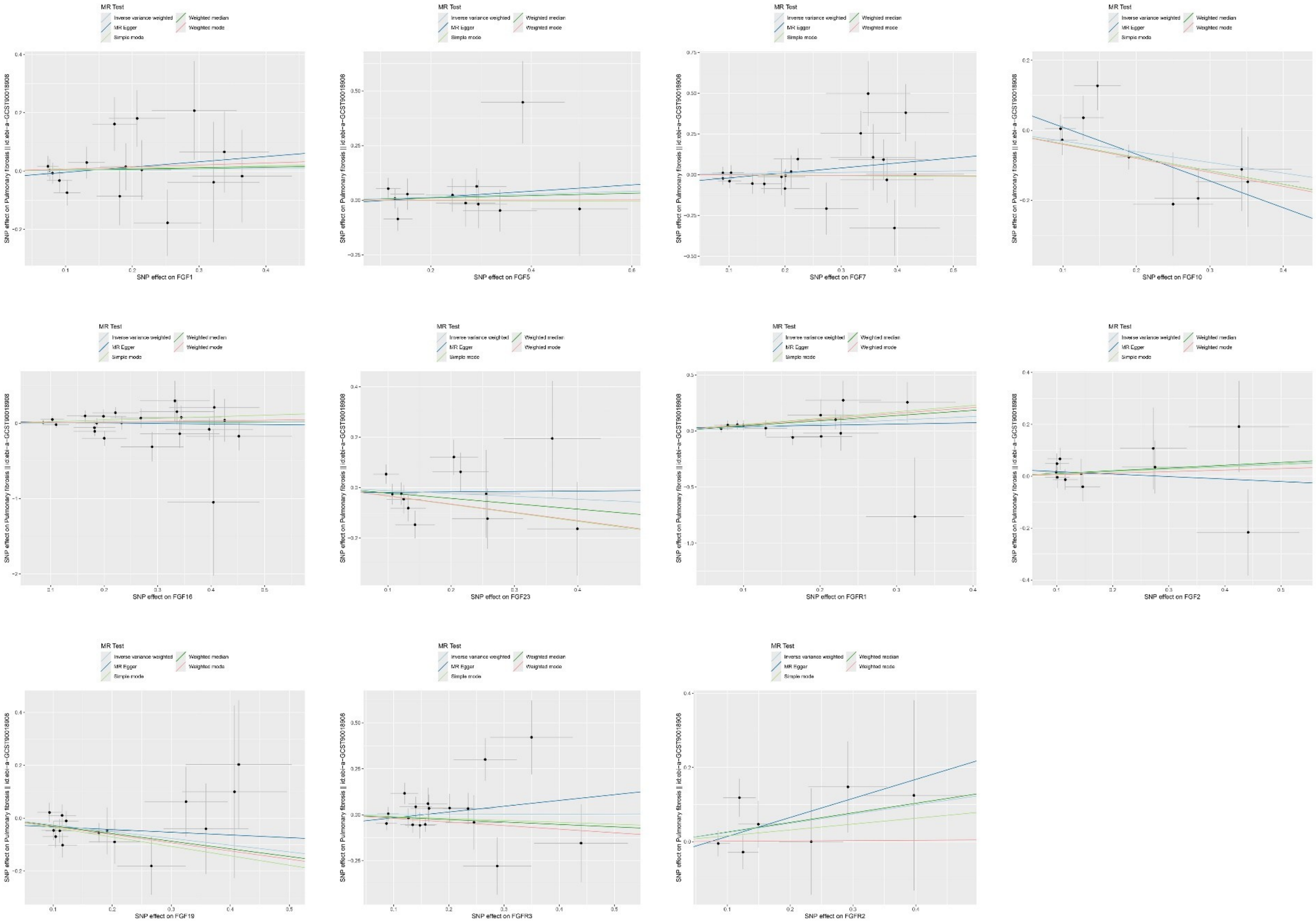

MR Test
Inverse variance weighted
MR Egger
Simple mode
Weighted median
Weighted mode
SNP effect on Pulmonary fibrosis || id:ebi-a-GCST90018906
SNP effect on FGF1
SNP effect on FGF5
SNP effect on FGF7
SNP effect on FGF10
SNP effect on FGF16
SNP effect on FGF23
SNP effect on FGFR1
SNP effect on FGF2
SNP effect on FGF19
SNP effect on FGFR3
SNP effect on FGFR2

Supplementary Figure S4. Scatter plot of the causal association between fibroblast growth factors (FGFs) and pulmonary fibrosis. This figure presents the scatter plots from the Mendelian randomization (MR) analysis evaluating the causal relationship between FGFs and pulmonary fibrosis. The x-axis represents the genetic effect of each single nucleotide polymorphism (SNP) on FGFs. The y-axis represents the genetic effect of the SNP on pulmonary fibrosis. Different MR methods, including inverse variance weighted (IVW), MR-Egger, weighted median, and weighted mode, are shown as regression lines to estimate the causal effect. Scatter plots are shown for FGF1, FGF5, FGF7, FGF16, FGF23, FGF2, FGFR3, and FGFR2. The direction and consistency of the regression lines indicate the presence or absence of a causal relationship between each FGF and pulmonary fibrosis, with potential pleiotropy assessed by the divergence of the MR-Egger regression line.
